# Investigating the Deterioration of Pavement Skid Resistance Using an Accelerated Pavement Test

**DOI:** 10.3390/ma16010422

**Published:** 2023-01-02

**Authors:** Xiaosheng Wu, Qiwei Chen, Yanqing Li, Niya Dong, Huayang Yu

**Affiliations:** 1 Guangzhou Cheng’an Testing Ltd. of Highway & Bridge, Guangzhou 510420, China; 2 School of Civil Engineering and Transportation, South China University of Technology, Guangzhou 510006, China; 3 Guangzhou Road Research Institute Co., Ltd., Guangzhou 510420, China

**Keywords:** stone matrix asphalt mixture, skid resistance attenuation, kneading test, pressure-sensitive film, finite element simulation

## Abstract

Stone matrix asphalt (SMA) mixture has been widely used in pavement engineering for its preferable in-service performance. However, deterioration of SMA pavement in skid resistance is apparent under traffic loading. There remains lacking attention on skid resistance attenuation of SMA pavement, which in turn is important for skid durability design in practice. Hence, this study aims to perform a thorough investigation to reveal the skid resistance attenuation law of SMA pavement. Multiple types of SMA-13 mixtures prepared by different material designs were selected to conduct a kneading test that simulates real surface states of in-service SMA pavement. Pressure-sensitive film and a 3D laser scanner were utilized for evaluating anti-skid performance and skid durability. The finite element (FE) method is introduced to simulate vehicle braking distance for skid-resistance evaluation. The results show that skid resistance attenuation of SMA pavement consists of two stages: In the first stage, the skid resistance of SMA pavement experiences a short enhancement, followed by a long-term weakening stage. Abundant surface texture of SMA helps to mitigate the impact of traffic load on skid resistance. The FE analysis and pressure-sensitive film results demonstrate the potential of skid durability design of SMA pavement based on the skid resistance attenuation law.

## 1. Introduction

Skid resistance is an important factor that influences the safety of driving on asphalt pavement. It is a property that depicts the pavement texture and friction behaviors when the pavement is subjected to tire load [1]. Related studies have validated that traffic accidents frequently happen when the road is of poor anti-skid performance [2,3,4,5]. About 30% of traffic accidents are caused by the lack of pavement skid resistance [6]. Therefore, growing concerns have focused on the skid resistance of the asphalt pavement currently to ensure the safety of vehicles driving on roads. Notably, SMA pavement typically performs well in skid resistance as it presents abundant forms of surface texture [7,8]. However, all types of asphalt pavement, including SMA pavement, inevitably experience skid resistance attenuation with the repeated vehicle loading after opening to traffic. It is of great significance to study skid resistance deterioration and skid durability of SMA mixtures.

Skid resistance results from the interaction between tire rubber and pavement material, providing sufficient friction force for vehicle tires. From the perspective of pavement, many factors such as aggregate hardness, angularities, and surface texture influence the anti-skid performance, together with the material design of the asphalt mixture [3,9]. Among them, the pavement surface texture plays an important role in the tire–pavement contact characteristics, which ultimately relates to the skid resistance of pavement [10]. Four scales of texture are categorized according to wavelength deviations [1]: the mega-texture, the macro-texture, the micro-texture, and the unevenness. Different texture scales vary contributions to the skid resistance of pavement. What primarily affects the skid resistance of pavements are micro-texture and macro-texture. Micro-texture refers to the microscopic structure on the coarse aggregate surface in the asphalt mixture. Micro-texture predominantly contacts the tire tread at molecular scale, providing skid resistance between the tire–pavement surface [11]. On newly layered pavements, aggregates exposed on the surface are masked by a binder film, which hinders the anti-skid effect of the micro-texture [12]. After opening to traffic, skid resistance tends to enhance temporarily due to the wearing of the binder film. Over long-term traffic loading, the micro-texture of aggregates is gradually polished due to repeated friction between the rolling tire and the pavement surface [13]. During this period, macro-texture serves as the main force to resist the tire–pavement friction. Macro-texture generally forms various grooves on the surface, which dominates the skid resistance at high vehicle speed [14]. Furthermore, deep and large macro-texture would create the fluent drainage of pavement, helping to increase the friction at wet tire–pavement interface [15]. However, macro-texture progressively weakens through time as the aggregates embed in the asphalt matrix, resulting in the decrease in average texture depth [16].

Under long-term traffic loading, the skid resistance of asphalt pavement cannot be efficiently guaranteed [2]. However, there has not been adequate attention on the attenuation and durability of skid resistance from the perspective of the tire–pavement interface. Continuous efforts have been devoted to developing laboratory tests to simulate the field pavement state under vehicle loads. Several accelerated tests were designed to imitate the deterioration of pavement skid resistance for predicting the anti-skid performance of terminal pavement state in field conditions under repeated traffic loading [17,18,19]. Wang [2] proposed the accelerated kneading machine to achieve an abrasion effect on exposed coarse aggregates along with compaction and reorientation of surface materials, representing the practical condition of in-service pavements. Traditional ways to characterize the skid resistance of asphalt pavement are texture measurements, which are widely used in numerous studies and pavement practices [20,21,22,23]. Researchers have contributed to predicting pavement friction coefficients for evaluating the effect of micro-texture on pavement skid resistance [1,24]. Frequently used testers for friction include the British pendulum tester (BFT), the dynamic friction tester (DFT), and the sideway force coefficient (SFC), among others. The effect of the macro-texture on pavement skid resistance depends on the wavelength, shape, and height of bulge on the surface. The representative indicators, mean profile depth (MPD) and mean texture depth (MTD), are popularly adopted to correlate with the skid resistance [6]. However, constraints of using macro-texture to predict skid resistance in field conditions were reported in some studies [25,26]. Skid resistance of pavement is a multi-parametric property affected by a number of factors. The liaison between measured texture and skid resistance appears to be inapplicable under different weather conditions [26].

The contact stresses emanated at the tire–pavement interface are important and closely linked with the skid resistance of pavement [3]. However, current research rarely takes into account the tire–pavement interfacial contact characteristics. Tire–pavement contact interface testing mainly focuses on two aspects: stress distribution pattern and surface texture characterization. Anghelache [27] inserted pressure sensors into the pavement surface to measure the magnitude and distribution of grounding stresses. Sakai [28] found that the light absorption at measurement points on the contact interface presents a specific function of contact pressure, which can be converted to contact pressure. Nevertheless, traditional pressure plate and light absorption methods appear to have low accuracy and low durability [2]. To accurately evaluate the skid resistance, pavement–tire interaction should be taken into consideration, where the tire characteristic is significant. The FE method is a promising choice to accomplish this task. Several finite element attempts have been made to predict the tire–pavement interactions and effectively evaluated the pavement skid resistance [29,30]. Coupling with the effect of water on skid resistance, a hydroplaning FE model can be easily constructed to analyze the tire–pavement interactions for a wet condition [31]. In view of pavement texture acquisition, Zhang [4] introduced a 3D dense-point scanning technology to obtain topography data of the specimen and printed 3D images of surface graphics. In addition, using 3D scanning technology to convert real texture information to the coordinates data needed for constructing FE pavement model has shown great potential [29,32]. The aforementioned test methods have provided a firm basis for the evaluation of pavement skid resistance deterioration. SMA pavement is widely used currently for its excellent anti-skid performance, durability, fatigue, and rutting resistance [33,34]. In terms of pavement lifecycle, the deterioration of skid resistance is inevitable when subjected to traffic loading. Even though the abundant surface texture and skeleton structure help to facilitate the skid resistance of SMA pavement, the attenuation of skid resistance remains a problem to be solved [6].

Based on the research described above, there is still lacking attention on the skid resistance deterioration of SMA pavement under traffic loading, which is in turn important for skid durability design in practice. To address the issue, this paper proposes a thorough investigation to reveal the anti-skid deterioration of SMA pavement. In particular, multiple types of SMA-13 mixtures prepared by different material designs are selected in this study. An accelerated pavement test is adopted on SMA specimens to simulate the actual pavement surface states under traffic loading. Further, pressure-sensitive film and a 3D laser scanner are utilized to characterize the anti-skid performance and skid durability of SMA pavement. Additionally, 3D-surface graphics data are used to establish the pavement model. FE analysis is introduced to simulate the actual vehicle tire driving on pavement, helping to reveal the skid resistance deterioration of SMA pavement.

## 2. Materials and Methods

### 2.1. Materials

**Figure 1:**
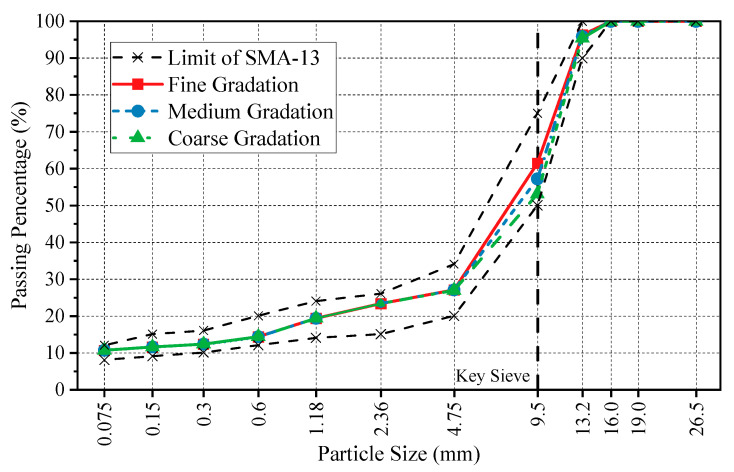
Grading curve for three types of SMA-13.

Modified styrene-butadiene-styrene (SBS) and granite aggregate were used to manufacture asphalt mixture and SMA specimens. The basic properties are shown in Table 1 and Table 2. SMA mixes with three types of gradation were designed taking 9.5 mm as the key sieve size, as shown in Figure 1. Considering the effect of binder film thickness on skid resistance, three asphalt contents were employed. In accordance with the orthogonal method, 9 groups of specimens were prepared, as shown in Table 3. The mechanical performance of specimens is shown in Table 4.

Rutting specimens were prepared with compaction machines. Asphalt and aggregates were mixed for approximately 3 min to manufacture SMA mixtures. After mixing, the asphalt mixture was transferred to an iron mould and compacted for about 24 times by the compaction machine to meet the standard specimens. The dimensions of standard SMA specimens in this test were 300 mm × 300 mm × 50 mm.

### 2.2. Experimental Program

#### 2.2.1. Accelerated Pavement Test

To simulate the attenuation of pavement skid resistance, a self-developed kneading machine was used in the test. An international standard rut-meter was partially improved to configure the kneading machine. The whole track broad experiences the vertical load and lateral shear force simultaneously during kneading, leading to abrasion of micro- and macro-texture on the specimen surface. Therefore, it can effectively simulate the actual tire loading on asphalt pavement.

**Figure 2:**
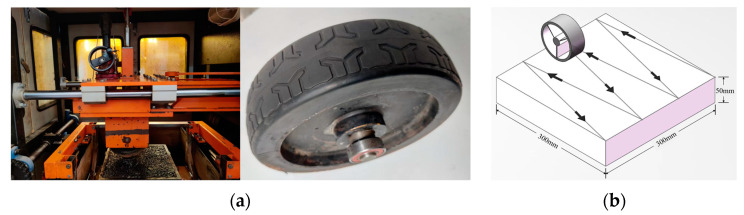
Accelerated pavement test: (**a**) kneading machine and tire and (**b**) tire kneading path.

The kneading is carried out by applying another motor which creates the tire’s reciprocating movement laterally while the original motor continues to drive the traction wheel in a longitudinal movement, as shown in Figure 2b. The research group has developed a set of kneading test systems [2]. In this study, the test was conducted at room temperature and humid condition. The lateral movement speed of the tire was 10 cm/min. The tire rolled 42 ± 1 times per min, and the wheel weight was adjusted to control the wheel pressure to 0.7 Mpa. A tread rubber tire was used in this test.

During the accelerated pavement test, the pavement specimens generated strain that cannot restore. Hence, the relationship between kneading time and actual pavement in-service time should be determined based on the accumulated damage. The accelerated loading study indicated that each accelerated load was equal to about 10.3 passes of standard axle load in terms of rutting and cracking behavior [35]. Regarding the abrasion effect, accumulated damage produced by the Aachen Polishing Machine is about 20 times more than standard tire wear [36]. On the basis of the prior experience [2,3,4], the relation of kneading time and actual pavement condition (design speed: 100 km/h, service level: 3, basic traffic capacity: 1600 pcu/(h·ln)) was predicted and shown in Table 5.

#### 2.2.2. Three-Dimensional Laser Scanning Test

To reveal the variation in specimen texture during the attenuation of pavement skid resistance, a 3D laser scanner was used in the test [4]. The micro- and macro-texture of the specimen was obtained with the horizontal and elevation data collected. During the measurement, several probes together collected distance information. Based on the principle of triangle measurement, coordinates of detecting points were obtained with an accuracy of 0.01 mm. Finally, the point cloud data were restructured in MATLAB to display 3D-surface graphics of the specimen. The displayed image range is 75 mm × 32 mm.

#### 2.2.3. Pressure-Sensitive Film Test

1. Mechanism of pressure-sensitive film technology

**Figure 3:**
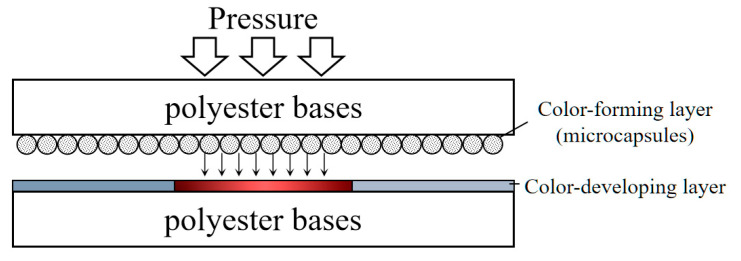
Pressure-sensitive film configuration.

Pressure-sensitive film comprises two polyester bases: a color-forming layer and a color-developing layer. When using the pressure-sensitive film, the two layers fit together. The color-forming layer carries the microcapsules, which will rupture and release color generation materials when applying pressure. Then, the developing materials within the color-developing layer interact with generation materials and turns dark red, as shown in Figure 3. According to the microcapsule sustained-release control technology, the darkness of red depends on the pressure applied.

In this test, the tire load was applied statically onto the pressure film for 2 min. Then, the color development film was taken out and left for 60 min until the color density was stabilized. The densitometer, a PerfectionTM V300 Photo CCD dedicated scanner, was used to describe the map of contact stress distribution with an image resolution of 0.125 (200 dpi). Finally, the numerical stress values were obtained by dedicated software (FPD-8010E). After processing and quantifying the pressure-sensitive film, information concerning the stress distribution was stored in a two-dimensional matrix:

(1) $$F(X,Y)=\left[\begin{array}{ccc} f(0,0) & \ldots & f(0,n)\\ \ldots & \ldots & \ldots \\ f(m,0) & \ldots & f(m,n) \end{array}\right]$$

where *F (X, Y)* is the overall contact stress (N) and *f (x, y)* is the single point contact stress (MPa).

2. Evaluation system of the pressure-sensitive film

The effective contact area refers to the area of all pressure response zones on the pavement surface under statically vertical tire load. The tire–pavement effective contact is in fact the squeeze contact between tread rubber and exposed aggregate structure of the pavement surface. It is believed that larger tire–pavement effective contact area represents better anti-skid performance of asphalt pavement [3].

The tire locally wraps coarse aggregates and produces embedded deformation, leading to stress concentration at the interface of tire and pavement. The skid resistance tightly correlates with the contact stress distribution. The stress concentration effect accelerates the abrasion of the pavement surface texture, which is adverse to the skid durability. To evaluate the stress concentration effect, the stress distribution concentration rate is introduced as follows:

(2) $$k_{f}=\frac{\iint_{D\prime }f_{\left(x,y\right)}dxdy}{\iint_{D}f_{\left(x,y\right)}dxdy}\times 100\%$$

where *k_f_* is the stress concentration rate, %; *f_(x,y)_* is the single point stress value in contact zone, MPa; and *D′* is the stress concentration area of tire–pavement contact, mm^2^. The area with stress above 1.8 MPa was determined according to the previous study, and *D* is the effective contact stress area, mm^2^.

#### 2.2.4. Finite Element Simulation

Recently, using the finite element method to establish tire–pavement coupling models has become a trend in revealing and evaluating the anti-skid performance of pavement. In this study, the tire–pavement contact model was established using ABAQUS finite element software. The braking distances were calculated as the indicator to evaluate the skid resistance of the pavement.

1. Tire modeling

A 175SR14 radial tire was adopted for the model. When characterizing the inflated tire, rubber was considered as superelastic material, and reinforcement was treated as isotropic elastic material. The construction of the tire’s geometric configuration was implemented as follows.

**Figure 4:**
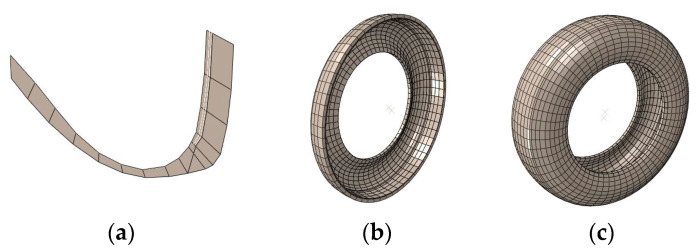
Tire modeling: (**a**) 2D half-section tire model; (**b**) partial tire model; and (**c**) complete tire model.

A 2D half-section tire model was firstly constructed in ABAQUS software. The plane model comprised the shoulder, tread, sidewall bead, and other parts of the tire. It was set as CGAX4. The rebar unit was employed to simulate the reinforcement composites of belt cord, carcass cord, bead wire, etc., the type of which was set as SFMGAX1. Then, the 2D model was rotated 360° around the circumference using the *SYMMETRIC MODEL GENERATION program provided by ABAQUS to obtain a partial 3D tire model (see Figure 4b). Finally, the partial model was axisymmetrically transformed to generate a complete 3D tire model (see Figure 4c). The completed 3D tire had 6962 nodes and 5283 C3D8R and SFM3D4R elements. The rubber material in the tire was defined as incompressible elements based on the Herrmann equation. Reinforcements used the corresponding reinforcing elements.

2. Construction of the tire–pavement contact model

The previously collected specimens’ texture information in this study was used for constructing the pavement model. Coordinates in the original 3D file were too dense and beyond the mesh density capability that ABAQUS can handle. To ensure the convergence of the contact model, a blunting process was adopted to eliminate the uneven phenomena of pavement coordinates. By repeating the trial calculations, the precision of the x–y mesh was finally selected to be 5 mm. The constructed pavement model was defined as the shell model. Transformed coordinates in the preprocessing INP file were visualized.

The original texture coordinates covered an area of 15×15 cm, which hardly simulated the actual movements of the tire in explicit analysis. Thus, the extension of the pavement model in rolling direction was conducted to provide enough tire running space. This process was subsequently realized by defining the mirror_matrix in MATLA. The coordinates of specimens were mirrored third times to extend to 120 cm in driving direction.

**Figure 5:**
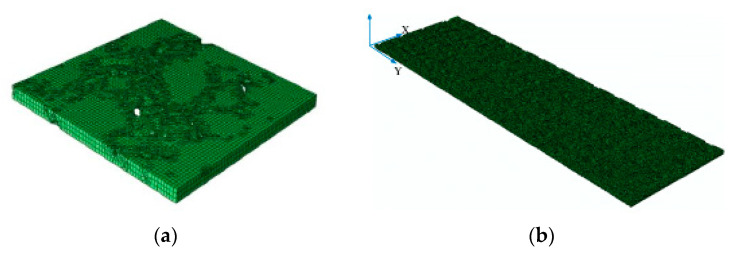
Pavement modeling: (**a**) SMA pavement model and (**b**) extended SMA pavement model.

In the explicit dynamic solver, the shell unit cannot block the water flow in the tire aquaplane model. Therefore, the simulation analysis generally takes the pavement units as analytic rigid bodies. The solid unit was used. The digital model of pavement texture processed by unit normalization and mesh passivation was extended along the *Z*-axis direction to obtain the 3D solid FE model, as shown in Figure 5.

Finally, the identifier S3 in the software was used to determine the type of pavement–tire contact model. The contact relation of tire, pavement, and fluid was defined by the universal contact algorithm. Then, the degrees of freedom for the bottom of the fluid model in the Z-direction and the two sides in the X-direction were constrained. A negative Y-direction velocity was applied to the water flow region with the same velocity as the pavement translation. The gravitational field was applied to the overall model in the Z-direction.

3. Construction of tire aquaplane model

In this study, the fluid–solid coupling effect was imported by FLUENT software. For the air–liquid phase flow issue, the VOF model (a surface tracking method under fixed Eulerian grids) was selected. In the model, both air and liquid components shared the same momentum equations. The volume rate occupied by each component was recorded within each calculation unit of the whole flow field.

Since FLUENT software only calculates the mechanical response of fluid at finite size, it is necessary to intercept the effective fluid calculation area. Based on the vertical stress and grounding characteristics, the fluid calculation area was determined. The dimensions were 1000 mm in the X-direction, which represented the tire forward direction; 500 mm in the Z-direction, which represented the tire width direction; and 40 mm in the Y-direction, which represented the fluid depth. The air thickness varied with water film thickness.

The calculation constrained the motion of the tire in translational direction and applied rotating angular velocity only. Additionally, translational velocity opposite to the forward direction was applied to the pavement and flow model simultaneously to simulate the tire driving on pavement water film. The front end of the fluid was set as the velocity inlet for fluid. The back end and the top were set as the pressure outlet. The left and right ends were set as frictionless walls, while the bottom was set as a moving wall.

**Figure 6:**
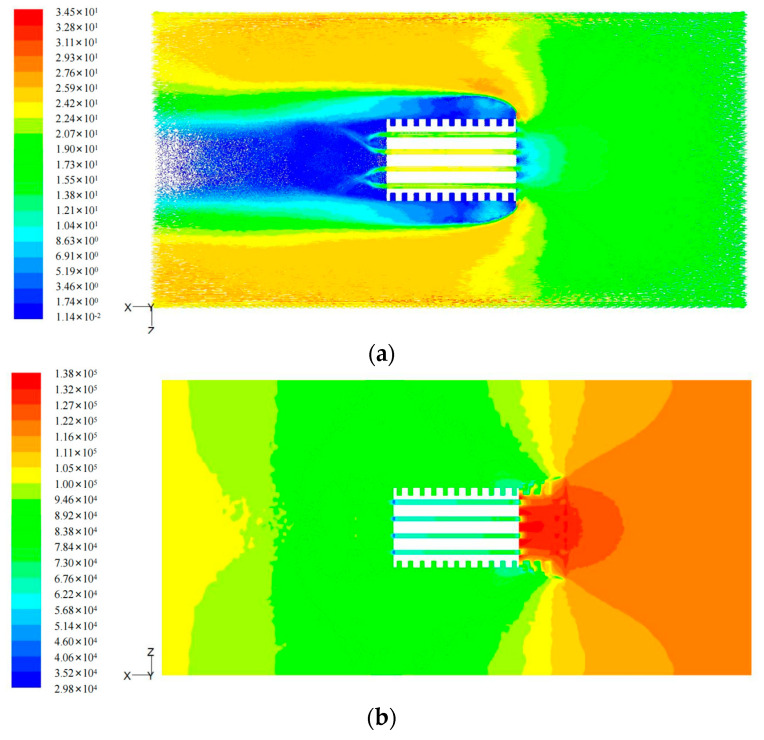
Water film condition (water film thickness = 6 mm, v = 75 km/h): (**a**) flow velocity distribution and (**b**) hydrodynamic pressure distribution.

When the tire rolled, the water film became wedge-shaped due to tire blocking, as shown in Figure 6a. Water formed a high-pressure area at the front of the tire, as shown by the red area in Figure 6b.

## 3. Results and Discussion

### 3.1. Attenuation of Stress Distribution

**Figure 7:**
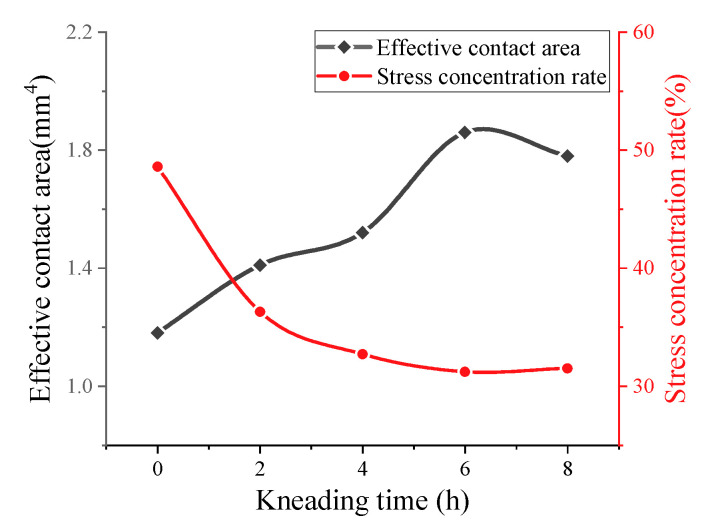
Attenuation of effective contact area and stress concentration rate.

**Figure 8:**
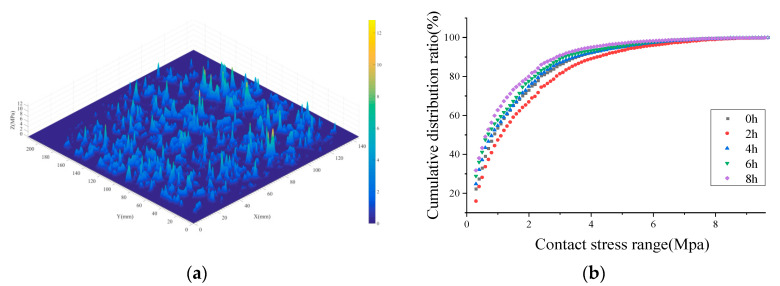
Attenuation of contact stress distribution: (**a**) initial contact stress distribution map and (**b**) cumulative contact stress distribution.

In the process of kneading, SMA pavement undergoes secondary compaction and abrasion of micro- and macro-texture. It is reflected in the attenuation of texture abundance and the change in tire–pavement contact area. Figure 7 and Figure 8 show the attenuation of effective contact area and stress distribution.

As shown in Figure 7, the effective contact area of SMA pavement continuously increases within the kneading time of 6 h. Kneading from 2 to 6 h, the effective contact area shows faster growth. During this period, coarse aggregates on the pavement surface cause a certain spatial rotation, leading to substantial fluctuations in the contact area. After 6 hours’ kneading, the surface coarse aggregates begin to stabilize and the pavement texture is mainly subjected to tire abrasion. The variation in contact area tends to be gentle and stable. For SMA mixes, the reconstruction of pavement texture occurs during 0–4 h kneading due to the rotation of coarse aggregates on the surface.

Figure 8 reveals the change in contact stress distribution. Initially, SMA pavement induces the stress concentration within a small area, creating a cone that is shown in the stress distribution map. With the kneading time increasing, the stress distribution curve changes from bottom to top, representing the increase in small stresses and the decrease in large stresses at the tire–pavement interface. In other words, kneading and test tires cause significant abrasion to the macro- and micro-texture of the SMA pavement. The pavement gradually becomes smooth and flat.

### 3.2. Morphological Changes in the Pavement Surface

**Figure 9:**
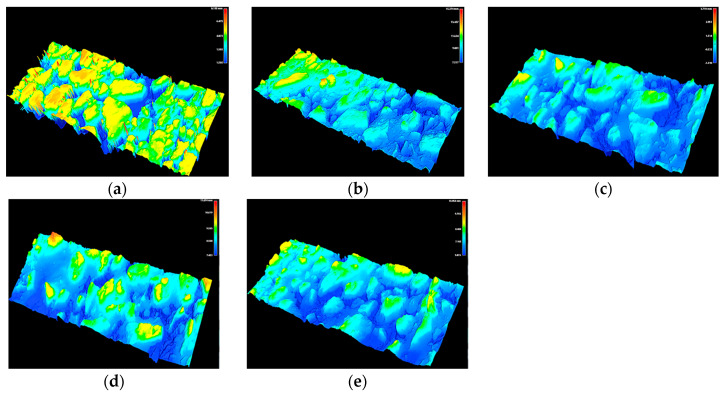
Morphological changes of surface texture: (**a**) kneading for 0 h; (**b**) kneading for 2 h; (**c**) kneading for 4 h; (**d**) kneading for 6 h; and (**e**) kneading for 8 h.

To reveal morphological changes in the pavement texture with skid resistance attenuation, a 3D laser scanner developed by South China University of Technology was introduced in this test. Micro- and macro-texture data for specimens were obtained and displayed as images with the help of MATLAB software. These data were also used in the later finite element analysis. After kneading for 0, 2, 4, 6, and 8 h, the changes in SMA-1 specimen texture are shown in Figure 9. In the service period for the asphalt pavement, aggregates experience the post-compaction of traffic, leading to a further degradation of the surface texture [13,16]. With the increase in kneading time, the attenuation of SMA pavement texture presents a pattern similar to that shown in the graphics. It is reflected in the secondary compaction of aggregates on the pavement surface and micro-texture abrasion effect. The initially sharp and rough texture of the SMA pavement gradually becomes smooth and rounded. As for SMA pavement, rich surface structure and skeletal effect offset the abrasion of traffic load to some extent, helping to improve the stability and durability of the anti-skid performance.

### 3.3. Skid Resistance Evaluation Based on Pressure-Sensitive Film

#### 3.3.1. Effective Contact Area

**Figure 10:**
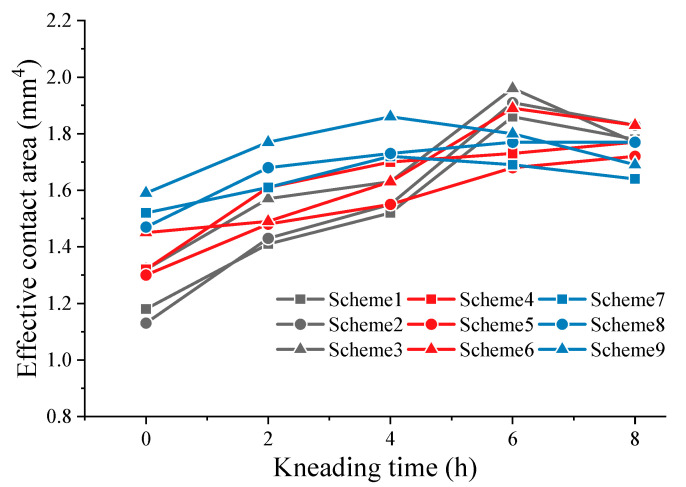
Results for effective contact area.

Figure 10 presents the results for effective contact area. The large variation in effective contact area of different SMA-13 specimens indicates the obvious effect of asphalt content and gradation on the skid resistance of SMA pavement. When the asphalt–aggregate ratio is 6.2%, the area of fine- and coarse-graded SMA pavement initially increases and then begins to decrease after kneading 4 h. Moreover, the attenuation trend is obvious (9.1% for coarse gradation and 4.7% for fine gradation). However, the effective contact area of intermediate graded pavement is relatively large and keeps increasing during kneading. Similar trends can be seen when asphalt–aggregate ratios of intermediate-graded SMA pavements are 5.8% and 6.0%. The effective contact area of all intermediate-graded SMA specimens is generally higher and most of them continue to increase during kneading. Therefore, intermediate-graded SMA pavements exhibit excellent anti-skid performance regardless of asphalt content.

To better evaluate skid durability of different SMA pavements, this study proposes the effective contact-area variation rate ΔA (shown in Equation (3)). When the result is above the “0” (positive value), it means that with the kneading time increasing, the anti-skid performance is growing. The low value of the result represents the slight growth in skid resistance but better anti-skid durability, and vice versa. When the result is below the “0” (negative value), the anti-skid performance is decaying; the larger the value, the more serious the anti-skid performance attenuation.

(3) $$\Delta A=\frac{A_{8}-A_{4}}{A_{4}}$$

where Δ*A* is the effective contact-area variation rate, %; A8 is the effective contact area kneading for 8 h, 10^4^ mm^2^; and A4 is the effective contact-area kneading for 4 h, 10^4^ mm^2^.

**Figure 11:**
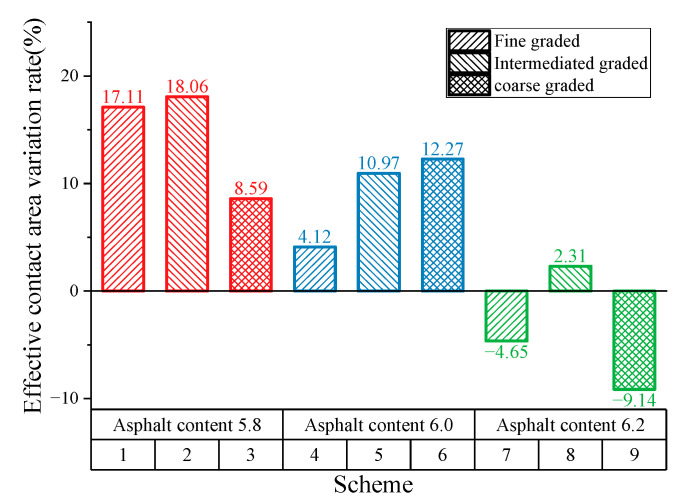
Results for effective contact-area variation rate.

As shown in Figure 11, both SMA pavements with 5.8 and 6.0 asphalt–aggregate ratios have positive ΔA values, indicating improved skid resistance. However, the above study shows that all SMA specimens with 5.8 asphalt–aggregate ratio display an obvious attenuation in the effective contact area. Specimens with 6.0 asphalt–aggregate tend to exhibit excellent anti-skid performance. Additionally, negative growth of the effective contact area occurs when the asphalt–aggregate ratio is 6.2. Kane [12] found that micro-texture of aggregates masked by binder film tends to lower the skid resistance. Inspired by the masking effect of the binder, it is clear that thicker binder film does more harm to the skid resistance of SMA pavement.

#### 3.3.2. Stress Concentration Effect

**Figure 12:**
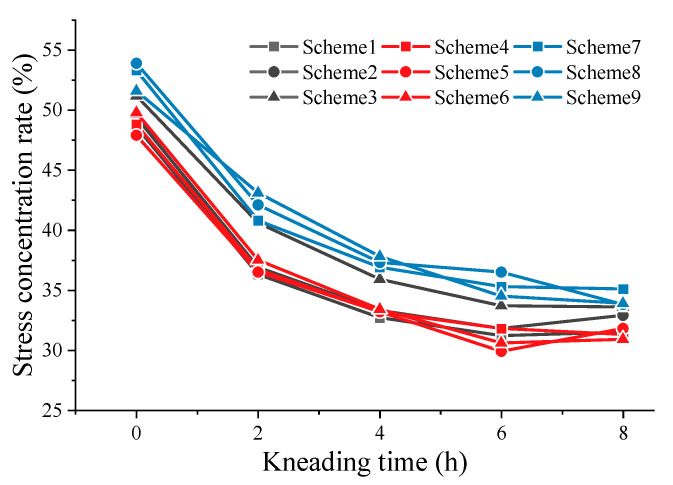
Results for contact stress concentration rate.

Figure 12 shows that contact stress concentration rate constantly decreases as kneading continues. Since specimens are subjected to the rolling of the tire in the early stage, macro-texture of specimens experience the secondary compaction leading to a reduction in stress distribution concentration rate. After kneading 4 h, binder film on the surface of aggregates is gradually eliminated, leaving the intact aggregate texture exposed on the surface. During this period, the anti-skid performance of SMA pavements is in the optimum state. Subsequently, the wearing tends to slow due to the abrasive effect of tires on pavement micro-texture, leading to the slowly descending stress concentration rate. It is also indicated that SMA pavements with 6.0 asphalt–aggregate ratio has the lowest concentration rate compared to that with other asphalt–aggregate ratios. Therefore, concerning skid durability, the optimal asphalt–aggregate ratio is considered to be 6.0. In addition, SMA pavements with high asphalt content have the worst skid durability.

The stress concentration variation rate ΔS (shown in Equation (4)) is introduced to evaluate the skid durability concerning the stress concentration effect.

(4) $$\Delta S=\frac{S_{8}-S_{4}}{S_{4}}$$

where Δ*S* is the stress concentration variation rate, %; S8 is the stress concentration rate for 8 h, %; and S4 is the stress distribution concentration rate for 4 h, %.

**Figure 13:**
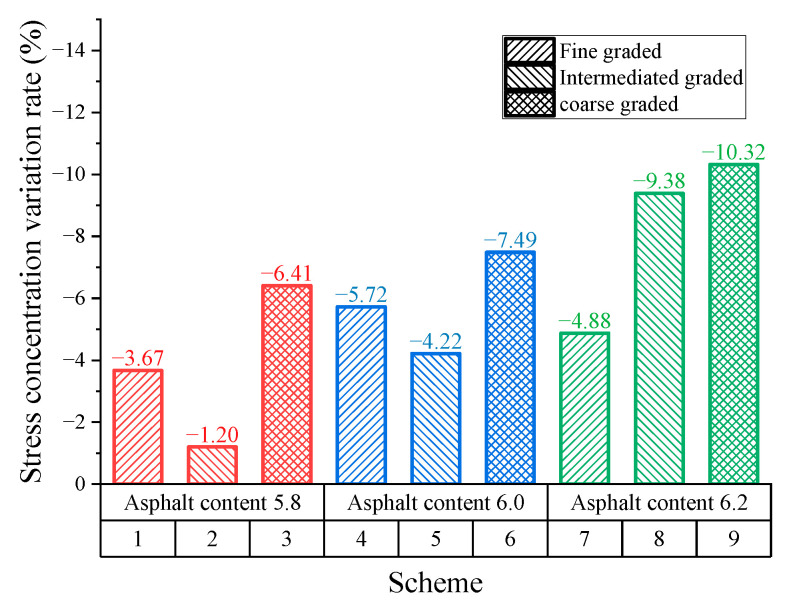
Results for contact stress concentration variation rate.

Figure 13 shows the results for the stress concentration variation rate. When the asphalt–aggregate ratio is 5.8, the intermediate-graded SMA pavement obtained the smallest variation rate for stress concentration (1.2%). This material design shows the best skid durability. The following is the fine gradation (3.67%) and the last is coarse gradation (6.41%). Similar results are obtained when the asphalt–aggregate ratio is 6.0. When the asphalt–aggregate ratio is 6.2, the variation rate for the fine-graded SMA specimen is at its minimum. However, its stress concentration rate remains the largest among all gradations, indicating the significant stress concentrate effect.

Previous studies [2,3,4] have confirmed that the continuously increasing tire–pavement contact area and low stress concentration effect is beneficial to skid resistance and skid durability of pavement. With the help of pressure-sensitive film, this section summarily determines the optimal skid durability design of SMA pavement: intermediate gradation through 9.5 mm key sieve and intermediate asphalt–aggregate ratio. In the construction of SMA pavements, it is recommended the asphalt–aggregate ratio be controlled at 6.0 ± 0.1 and the 9.5 mm sieve passage rate be controlled at 57% ± 2%. It is certain to have guaranteed anti-skid performance with this design method.

### 3.4. Subsection Skid Resistance Evaluation Based on Braking Distance Simulation

#### 3.4.1. Braking Distance in Dry Conditions

The calculated braking distances for dry conditions are shown in Table 6. The braking distance of SMA surface increases with the initially increasing speed and decreases with the increasing braking deceleration speed. When initial speed and deceleration speed is certain, the simulated braking distance firstly increases in short and then decreases constantly with increasing kneading time. This phenomenon can be explained as follows. The SMA mixture is made of rich asphalt mastic, coarse aggregate skeleton, and fine aggregate filling. At the beginning of the kneading test, fine aggregates are worn off and the coarse aggregate edges are polished. Subsequently, the friction of pavement–tire interface decreases, leading to the increase in braking distance. With the kneading continuing, coarse aggregates start to become the main force for skid resistance. It is reported that the macro-texture formed by coarse aggregates determines the skid resistance on the early stage of pavement service [12,37]. The angularity of aggregates is gradually smoothed and tends toward stability in appearance. This results in a continuous increase in frictional resistance and a consequent decrease in braking distance.

**Figure 14:**
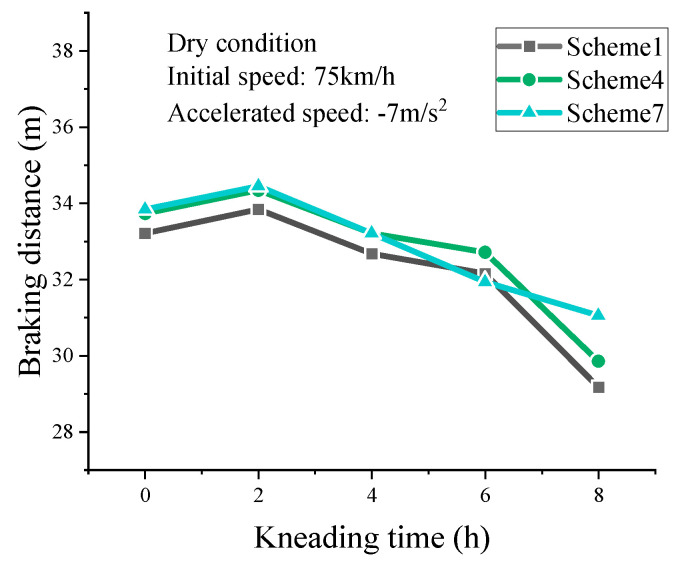
Results for braking distance in dry conditions.

Since serious traffic accidents most commonly occur when vehicles drive at high speed, braking distances with 75 km/h initial speed and −7 m/s^2^ deceleration speed are selected, as shown in Figure 14. As the kneading time increases, the braking distance on SMA pavement increases at first and then decreases constantly for the three schemes. In the terminal state (kneading for 8 h), the simulated braking distance for Scheme 1 and Scheme 4 show a significant decreasing trend while the decreasing trend for Scheme 7 is not apparent. The simulated braking distance for Scheme 7 in its terminal state is the largest compared to other schemes. Therefore, the high asphalt–aggregate ratio is very unfavorable for the skid resistance of SMA pavement in dry condition.

#### 3.4.2. Braking Distance in Wet Conditions

As shown in Table 7, braking distances on wet pavement also increase with the initial speed increasing and decrease as the deceleration speed increases, which is the same as the braking distance in dry conditions. However, the braking distance in wet conditions is relatively greater. It is because the water film serves as a lubricant film between the tire and pavement surface, reducing the frictional resistance. Compared to the results in dry conditions, the presence of water film has little effect on the braking distance with low initial speed. However, high initial speed appears to significantly increase the braking distance. With continued kneading, the braking distance is tending to rise initially and then fall constantly. The cause of this phenomenon is the same as that in dry conditions.

**Figure 15:**
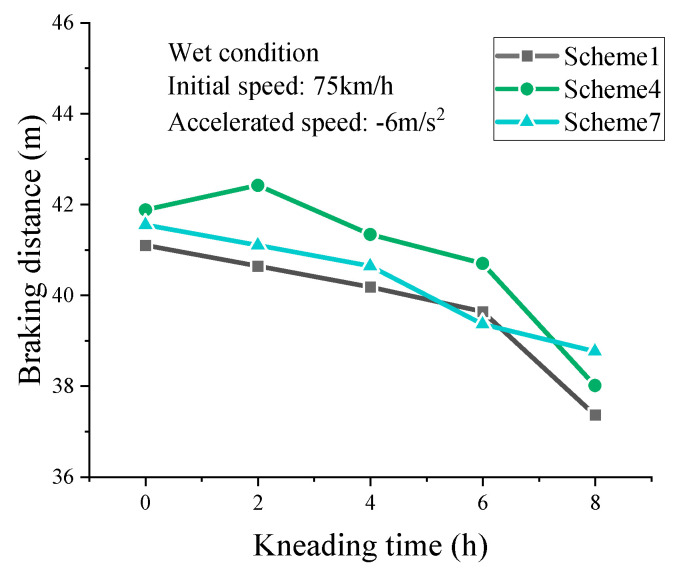
Results for braking distance in wet conditions.

In wet conditions, the high-speed braking of vehicles shows more serious hazards. The actual braking deceleration speed does not reach −7 m/s^2^ on wet pavement. Thus, a braking initial speed of 75 km/h and a deceleration speed of −6 m/s^2^ are selected for analysis. As shown in Figure 15, braking distance of SMA-13 for three asphalt contents show the trend of initial increase and then constant decrease as kneading continues. The simulated braking distance of Scheme 7 in the terminal kneading state is the largest. The result shows that high binder film thickness has a detrimental effect on the skid resistance of SMA pavement. In addition to skid resistance, the binder film thickness also influences the mechanical properties of asphalt pavement. Therefore, skid resistance can be regarded as a parameter in the material design of SMA mixture.

## 4. Conclusions

In this study, the actual surface states of SMA pavement were simulated by an acceleration pavement test. The stress distribution of tire loading on SMA pavements was tested using pressure-sensitive film. Together with the finite element method, the deterioration of SMA skid resistance was evaluated. The findings can be summarized as follows:

(1) The skid resistance attenuation of SMA pavement consists of two stages. In the first stage, the skid resistance experiences an enhancement because of the aggregates’ secondary compaction and polishing of attached binder on the surface. Subsequently it experiences a long-term weakening stage due to exposed aggregates being polished. However, abundant surface texture of SMA mitigates the impact of spatial fluctuation in surface coarse aggregates.
(2) The pressure-sensitive film test system effectively evaluated the contact stress distribution and effective contact area at the tire–pavement interface. SMA-13 with high binder content resulted in poor anti-skid performance and skid durability. However, SMA pavement generally suffers from poor performance for a low amount of binder. Therefore, SMA-13 with a 6.0% asphalt–aggregate ratio and intermediate gradation through 9.5 mm key sieve is much more favorable concerning comprehensive performance.
(3) Numerical simulation analysis showed that a thick binder film is unfavorable to the skid resistance of SMA pavement. Based on the attempts in this study, it would be of great practical significance to characterize the attenuation of pavement skid resistance using the accelerated pavement test and FE method.

Based on the accelerated pavement test, this study investigated the deterioration of pavement skid resistance in terms of tire–pavement interfacial contact characteristics. The pressure-sensitive film system effectively evaluated the attenuation pattern of pavement skid resistance. In parallel, the FE method was adopted to calculate the braking distance under tire driving to characterize skid resistance. However, the pavement surface simulated in this paper is a horizontal surface, which differs from the actual surface state. To simplify the complexity of the pavement model, micro-texture is not considered in the model, which is essential for actual pavement skid resistance. These issues will be further explored in future studies.

## Figures and Tables

**Table 1:** Technological parameters of the SBS-modified asphalt.

Properties		Specification Requirement	Test Results
Penetration 25 °C, 100 g, 5 s (0.1 mm)		40–60	53
PI	Min	0	0.6
Ductility 5 °C, 5 cm/min (cm)	Min	20	29
Softening point *T_R&B_* (°C)	Min	60	85.3
Flash point (°C)	Min	230	279
Solubility (%)	Min	99	99.6
Elastic recovery 25 °C (%)	Min	75	94
Kinematic viscosity (Pa s)	135 °C Max	3	2.56
	165 °C	-	-
RTFOT residue(163 °C, 85 min)	Quality variation (%)	± 1.0	−0.12
	Ductility 5 °C (cm) Min	15	40.2
	Penetration ratio (%) Min	65	78.8

**Table 2:** Basic properties of aggregates.

Properties	Unit	Test Results
Apparent density	g/cm^3^	2.670
Crushing value	%	14.5
Abrasion	%	18.0
Water absorption	%	0.38
Friction coefficient (BPN)	%	41.3

**Table 3:** Material designs of SMA-13 specimens.

Scheme No.	Asphalt Content (%)	Material Designs
1	5.8	Low binder and fine gradation
2		Low binder and medium gradation
3		Low binder and coarse gradation
4	6	Medium binder and fine gradation
5		Medium binder and medium gradation
6		Medium binder and coarse gradation
7	6.2	High binder and fine gradation
8		High binder and medium gradation
9		High binder and coarse gradation

**Table 4:** Mechanical performance of SMA-13 specimens.

Scheme No.	Air Voids (%)	VFA (%)	VMA (%)	Stability (KN)	60 °C Dynamic Stability (Times/mm)	TSR (%)	Leaking Loss (%)	Cantabro Loss (%)
1	3.4	79.5	16.8	9.22	7328	87.6	0.04	3.0
2	3.7	78.1	16.9	9.53	7463	89.2	0.05	3.6
3	3.9	76.6	17.1	9.86	7639	90.1	0.07	4.1
4	3.2	81.3	16.6	9.47	7436	88.5	0.05	2.7
5	3.5	79.4	16.7	9.79	7601	90.3	0.06	3.2
6	3.8	77.3	16.9	9.98	7789	91.0	0.08	3.8
7	3.0	82.6	16.5	9.53	7586	89.6	0.06	2.3
8	3.4	80.7	16.6	9.86	7767	90.8	0.07	2.9
9	3.6	79.4	16.7	10.11	8013	91.5	0.09	3.6

**Table 5:** Predicted relation of kneading time and actual pavement condition.

Kneading Time (h)	Predicted Actual Condition
0	Open to traffic initially
2	Open to traffic for 4–6 months
4	Open to traffic for 9–12 months
6	Open to traffic for 15–18 months
8	Open to traffic for 20–24 months

**Table 6:** Results for braking distance on dry pavement (m).

Accelerated Speed (m/s^2^)	−5			−6			−7		
Initial Speed (km/h)	25	50	75	25	50	75	25	50	75
Scheme 1 (0 h)	6.01	20.92	45.09	4.97	17.53	38.46	4.4	14.02	33.21
Scheme 1 (2 h)	6.22	21.22	45.46	5.26	17.92	38.95	4.87	14.57	33.84
Scheme 1 (4 h)	5.86	20.65	44.67	4.65	17.09	37.98	4.01	13.61	32.67
Scheme 1 (6 h)	5.55	20.27	44.29	4.31	16.64	37.41	3.59	13.16	32.15
Scheme 1 (8 h)	5.21	18.84	41.82	4.36	16.12	35.01	3.82	12.25	29.17
Scheme 4 (0 h)	6.06	20.94	45.72	5.01	17.98	38.81	4.41	14.07	33.73
Scheme 4 (2 h)	6.27	21.21	46.09	5.24	18.36	39.29	4.90	14.63	34.34
Scheme 4 (4 h)	5.91	20.70	45.29	4.76	17.56	38.34	4.01	13.65	33.21
Scheme 4 (6 h)	5.61	20.36	44.90	4.49	17.12	37.78	3.58	13.19	32.71
Scheme 4 (8 h)	5.27	19.08	42.38	4.53	16.62	35.44	3.81	12.27	29.85
Scheme 7 (0 h)	6.22	21.22	45.46	5.26	17.92	38.95	4.87	14.57	33.84
Scheme 7 (2 h)	6.47	21.54	45.86	5.59	18.33	39.42	5.34	15.04	34.45
Scheme 7 (4 h)	6.01	20.92	45.09	4.97	17.53	38.46	4.4	14.02	33.21
Scheme 7 (6 h)	5.47	20.15	44.07	4.19	16.41	37.12	3.43	12.92	31.93
Scheme 7 (8 h)	5.32	20.27	44.94	4.45	16.39	36.48	3.81	13.43	31.05

**Table 7:** Results for braking distance on wet pavement (m).

Accelerated Speed (m/s^2^)	−5			−6			−7		
Initial Speed (km/h)	25	50	75	25	50	75	25	50	75
Scheme 1 (0 h)	6.51	21.61	46.04	5.47	19.64	41.10	5.39	18.01	38.24
Scheme 1 (2 h)	6.30	21.31	45.67	5.15	19.26	40.64	4.86	17.49	37.53
Scheme 1 (4 h)	6.15	21.03	45.25	4.78	18.83	40.18	4.43	17.10	36.92
Scheme 1 (6 h)	5.83	20.65	44.88	4.40	18.39	39.64	3.96	16.67	36.33
Scheme 1 (8 h)	5.48	19.19	42.43	4.46	17.88	37.36	4.22	15.81	32.96
Scheme 4 (0 h)	6.57	22.46	46.69	5.57	20.05	41.88	5.38	18.27	38.29
Scheme 4 (2 h)	6.35	22.15	46.31	5.29	19.66	42.42	4.86	18.09	38.02
Scheme 4 (4 h)	6.20	21.86	45.88	5.01	19.23	41.34	4.31	17.25	37.67
Scheme 4 (6 h)	5.88	21.47	45.50	4.63	18.78	40.70	3.86	16.07	36.07
Scheme 4 (8 h)	5.52	19.97	43.00	4.69	18.26	38.01	4.11	16.41	34.65
Scheme 7 (0 h)	6.77	21.94	46.43	5.85	20.04	41.55	5.91	18.46	38.93
Scheme 7 (2 h)	6.51	21.61	46.04	5.47	19.64	41.10	5.39	18.01	38.24
Scheme 7 (4 h)	6.30	21.31	45.67	5.15	19.26	40.64	4.86	17.49	37.53
Scheme 7 (6 h)	5.75	20.52	44.66	4.26	18.16	39.36	3.79	16.44	36.08
Scheme 7 (8 h)	5.60	20.65	45.52	4.56	18.14	38.76	4.21	16.93	35.09

## Data Availability

The data presented in this study are available on request from the corresponding author.
